# Widening the Lens: Reflecting on the Mixing of System Dynamics With Action Research Alongside Work Within the Problem Structuring Methods Field: Comment on "Insights Gained From a Re-analysis of Five Improvement Cases in Healthcare Integrating System Dynamics Into Action Research"

**DOI:** 10.34172/ijhpm.2023.7620

**Published:** 2023-10-08

**Authors:** Fran Ackermann

**Affiliations:** School of Management and Marketing, Faculty of Business and Law, Curtin University, Perth, WA, Australia.

**Keywords:** Action Research, System Dynamics, Problem Structuring Methods, Learning

## Abstract

This commentary reflects upon the insights for improvement cases reported by Holmström et al where they consider the integration of action research (AR)—a research methodology—and system dynamics (SD)—a modelling technique—to manage the complexity of patient care pathways. Whilst this combination can be effective, recognising that SD is a simulation model whereas AR is a research approach is important for both practical and conceptual reasons. In addition, some of the benefits noted can also be achieved through taking a wider examination of modelling techniques, particularly problem structuring methods (PSMs) as SD has been considered a PSM and PSMs are designed to effectively engage multi-disciplinary group members in the search for solutions as this will provide further avenues for both engagement and learning.

 Holmström and colleagues^1^ present a strong case for using system dynamics (a continuous simulation modelling approach) within an action research (AR) setting to enable healthcare workers to develop more effective solutions. Building on a retrospective analysis of 5 case studies, the paper identifies that (*a*) groups move between convergence and divergence phases, (*b*) there is an increase in the likelihood of implementation, and (*c*) learning takes place through deep engagement and co-creation of action. This is clearly a valuable study.

 As such, this work builds on an existing body of extant work, particularly in the management science/operational research (MS/OR) arena. The need for, and use of, qualitative and quantitative modelling to support groups in tackling complex messy problems is well established. Models provide the opportunity to take a systemic approach (increasing the likelihood of sustainable outcomes), exploring how different views interconnect and how a shared language/understanding is created. In addition, models can take the form of a ‘negotiative device’^2,3^ facilitating socio-political considerations. Consequently, models can enhance the chances of implementation. System dynamics (SD) using causal loop diagrams (CLDs) provides the means for negotiating what the ‘problem’ is, and complemented by computer-based simulation for assessing the impact of actions on the system, realises these benefits. Moreover, using an AR approach when modelling has been seen to be an important part of the Operational Research world, both from a decision-making quality perspective as well as from a social-behavioural perspective.^4^ This commentary seeks to explore in more detail both aspects extending the contribution of the paper.

## Models for Learning, Negotiation, and Ownership

 The authors note early on that there are considerable challenges when bringing multi-professional staff together due to there being different knowledge bases and different power bases. Whilst the first of these is predominantly a content consideration, the second focuses on process issues. As such designing approaches that consider both process and content modelling can increase the likelihood of success. SD modelling, both through the construction of CLDs and the running of the simulation model not only enables the range of different views to be accommodated but also ensures that all participants are on the ‘same page.’ As such the CLD and/or simulation model can act as a transitional object^5^ and/or boundary object^6^ enabling the group to navigate both the process and content challenges. For example, models, whether they be SD-oriented or some other form, provide the means of separating the proponent from the contribution further helping to manage some of the socio-political considerations. Attending to both process and content is important as one can usefully inform the other acting as a ‘multiplier’ effect^7^ and understanding how best to embed this within decision-making in any realm is a worthy activity.

 The authors also touch on the role of learning when embracing SD modelling augmented with AR. Whilst this is not new as a range of authors for example Thompson et al^8^ and Peck^9^ report on this phenomenon, it adds to our understanding of how models can facilitate sense-making – a necessary requirement when working with complex problems such as healthcare provision. It would be interesting and useful to compare how the models built in the healthcare situations facilitated learning as compared with the experiences of other researchers. What new insights were there, and what lessons were to be learnt? This is particularly of interest given that the study focused on work that combined group discussion through workshops with backroom modelling – a practice used by researchers and consultants typically seeking to balance the complexity of SD modelling with time efficiency (recognising that the participants were time-poor). It would be interesting to consider whether this separation enables the most effective learning or whether a different balance would better enable those participating to learn and negotiate successful outcomes. This consideration is stimulated by the fact that Figure 1 shows different balances between facilitated group work and modeller backroom development throughout the cases with the beginning and end being dominated by facilitated group work, suggesting that it is not only the *balance* between the two modes of working but *where* in the intervention one mode appears to take precedence over another. It would also be interesting to consider whether the gaps between workshops allowed for participants to sound out some of the insights emerging from the workshops with others in their workplace – checking both the content considerations (for example was anything missing or erroneous) along with the process considerations (ensuring political feasibility for emergent outcomes) – a feature that has been noted by Ackermann et al.^10^

 The paper additionally raises the value of co-creation increasing the likelihood of implementation. This benefit has been noted in strategy research whereby co-created models enable participants to gain both emotional and cognitive commitment to the outcomes as well as enhance their understanding of the problematic situation.^11^ Understanding better how to facilitate co-creation is important as it attends to both process and content management and thus teases out the activities that prove to be particularly efficacious adding to the extant knowledge. Augmenting this would be considering explicitly how best to ensure that the process embedded principles of procedural justice.^12^ The paper notes the value of engagement and explanation – two of the 3 principles proposed by Kim and Mauborgne^12^ and potentially touches on the third expectation, clarity, adding further flesh to the principles. Moreover, through further reflection on the cases, it might be possible to identify which activities contributed to attending to the principles taking note of whether there were any healthcare particularities – recognising that in some instances, context matters.

 One of the aspects I found challenging was that there was little recognition of the work done in the field of problem structuring methods (PSMs).^13^ PSMs explicitly address situations where there is not an agreed problem definition, where the ‘problem’ is disputed – a characteristic that was noted as being present in the case studies. PSMs also support the identification of cause and effect seeking the root causes as does CLD. They attend to both the process and content considerations and strongly advocate for facilitation. Finally, they too attend to sense-making and shared mental models. Some consideration as to how this study builds, elaborates, or contradicts findings in the PSM literature would extend our knowledge of how best to tackle complex problems in healthcare and beyond.

## Mixing Methods – Moving Beyond Modelling Methods

 The paper notes that one of its contributions lies in its integration of SD modelling with AR noting that this constitutes a mixed methods approach. This assertion raises two important considerations. The first is that the mix moves beyond the established concept of mixed methods referred to. For example, the paper cites Mingers and Gill^14^ noting that ‘no single methodology can offer a complete view of the complexities facing organisations’ however, Mingers and Gill focus on mixing MS/OR methods whereas the paper views mixing methods as combining modelling techniques with research approaches. As such further reflection on this form of mixing of methods would be worthwhile, both from a conceptual perspective as well as a practical one.

 Furthermore, the combination touches on the second consideration which is the assertion that using a modelling technique alongside AR is new. This is not the case as illustrated by Peter Checkland’s work where AR is explicitly mentioned as being a key component. For example, it is noted by Rose^15^ that “Soft systems methodology is well established as a vehicle for action research, particularly in programmes initiated at Lancaster University.” Other work in the PSM field also operates within the AR approach. How then is the approach presented in the paper adding to our knowledge of using models within an AR approach? For example, what activities/approaches worked well in healthcare where there are multiple stakeholders with vastly different knowledge bases and power hierarchies?

 Another area where further exploration would provide value is how the research avoided crossing the fine line between consultancy and AR.^16^ It has been argued that to ensure robust AR it is necessary to have a detailed pre-understanding of the methods and tools, allowing for operationalisation and subsequently reflection. Given that this study centres on a re-analysis of cases it is not clear whether a clear design had been produced in advance and subsequently reflected upon. More detail here would help further our understanding of the use of AR from a retrospective stance and enhance our understanding of where, when, and how AR can be undertaken. Given that the research is based on an analysis of 5 cases, how do the insights elicited from the scrutiny of these cases map onto the AR cycle for increased robustness? For example, were there any lessons from the first case study employed when undertaking the subsequent studies? Finally, given the authors used James and colleagues’^17^ work to understand the cases, and centres on how to use and choose a management consultant (and is in the business literature rather than academic literature) what impact might this have had on the outcomes?

## Discussion

 The paper presents further evidence for the use of modelling *with* groups – seeking to develop solutions that are both procedurally rational as well as procedurally just – extending our knowledge and bringing the integration further into the health discipline. As such it provides valuable insights. However, it appears to take a slightly myopic view as it does not consider related literature that would help both in terms of extending our knowledge of visual interactive modelling and bringing disparate disciplines together. That said, it is recognised that this is a challenge for any researcher. Extrapolating into the future, it might also be worth considering how developments in artificial intelligence (for example generative artificial intelligence) could not only help in the development of models (for example, ChatGTP is able to produce excellent software code) but also in the sourcing of relevant information – both as an input into the SD model but also as a means of integrating the SD modelling with other complementary models (potentially giving rise to further insights and deeper understanding). This could be augmented with the improvements being made in online processes enabling (*a*) appropriate (in terms of being able to access all the right people) engagement, and (*b*) potentially bringing together the backroom work and workshops facilitating learning. To conclude, the research provides an alternative way of considering mixing methods from that presented within the MS/OR world and encompasses the mixing of OR methods, to one that integrates modelling techniques with research methods broadening the concept of mixing methods and opening avenues for further research into wider combinations.

## Ethical issues

 Not applicable.

## Competing interests

 Author declares that she has no competing interests.
